# Experimental Study of Thoracoabdominal Injuries Suffered from Caudocephalad Impacts Using Pigs

**DOI:** 10.1155/2018/2321053

**Published:** 2018-05-10

**Authors:** Sishu Guan, Zhikang Liao, Hongyi Xiang, Xiyan Zhu, Zhong Wang, Hui Zhao, Peng Liu, Xinan Lai

**Affiliations:** ^1^Department of Spine Surgery, Daping Hospital, Third Military Medical University, Chongqing 400042, China; ^2^State Key Laboratory of Trauma; Burns & Combined Wound, Institute for Traffic Medicine, Third Military Medical University, Chongqing 400042, China

## Abstract

To know the caudocephalad impact- (CCI-) induced injuries more clearly, 21 adult minipigs, randomly divided into three groups: control group (*n* = 3), group I (*n* = 9), and group II (*n* = 9), were used to perform the CCI experiments on a modified deceleration sled. Configured impact velocity was 0 m/s in the control group, 8 m/s in group I, and 11 m/s in group II. The kinematics and mechanical responses of the subjects were recorded and investigated. The functional change examination and the autopsies were carried out, with which the injuries were evaluated from the Abbreviated Injury Scale (AIS) and the Injury Severity Score (ISS). The subjects in group I and group II experienced the caudocephalad loading at the peak pelvic accelerations of 108.92 ± 58.87 g and 139.13 g ± 78.54 g, with the peak abdomen pressures, 41.24 ± 16.89 kPa and 63.61 ± 65.83 kPa, respectively. The injuries of the spleen, lung, heart, and spine were detected frequently among the tested subjects. The maximal AIS (MAIS) of chest injuries was 4 in group I and 5 in group II, while both the MAIS of abdomen injuries in group I and group II were 5. The ISS in group II was 52.71 ± 6.13, significantly higher than in group I, 26.67 ± 5.02 (*p* < 0.05). The thoracoabdomen CCI injuries and the mechanical response addressed presently may be useful to conduct both the prevention studies against military or civilian injuries.

## 1. Introduction

The injuries induced by caudocephalad impacts (CCI) frequently occurred in military vehicles, for example, underbelly blasts (UBB), which have led to large numbers of injuries and deaths [1–3]. Some civilian accidents, such as helicopter crashes and falls [4–11], resulted in also so many casualties owing to the CCI. For example, helicopters are being widely used worldwide nowadays due to their excellent motorization, with an estimated incidence of 2.5 helicopter crashes per 100,000 flying hours [9], and the mortality rate of the injuries involved in helicopter crashes was up to 59% [5]. Falls contributed to approximately 35 million disabilities or adjusted life years annually, showing an increase trend [11].

Thoracoabdomen is vulnerable to the CCI, and the CCI-induced thoracoabdominal injuries were reported frequently, with potential disaster outcomes [12–16]. As compared to the thoracoabdomen injuries induced by the horizontal impacts, such as anterior-posterior or lateral, remarkable discrepancies existed in injury mechanism, injury characteristics, and injury tolerance from the injuries by the CCI. In the past decades, the reported researches with regard to CCI-related injuries were done by retrospectively analyzing the injuries or deaths involved in the military or civilian scenarios [12–16] or performing the impact experiments with a dummy or cadaver [17–22]. The results have played key roles in understanding the injury mechanisms and evaluating the injury response for the CCI injuries, that is, fractures for the spine. However, the studies using postmortem human surrogates (PMHS) or finite element analysis (FEA) hardly produced successfully the injuries by thoracoabdominal organs frequently reported in military or civilian scenarios, particularly hemorrhage, and as a result, the injuries led by the CCI remained unclear. Furthermore, there still has been a paucity of experimental data concerning the thoracoabdominal CCI injuries until now, especially with animals.

In order to more clearly address the thoracoabdomen CCI-induced injuries, adult minipigs, whose anatomical structures of thoracoabdominal organs and physiological changes of the impact injury are similar to human body to some extent [23], were employed as a surrogate model of human body in the present study.

## 2. Material and Methods

This study was carried out strictly following the recommendations in the Guide for the Care and Use of Laboratory Animals of the National Institutes of Health. The protocol was approved by the Animal Ethics Committee of the Third Military Medical University (Permit number: 20150402), and great efforts were made to minimize suffering of the experimental animals and cut down the animal number.

### 2.1. Experimental Setup

**Figure 1:**
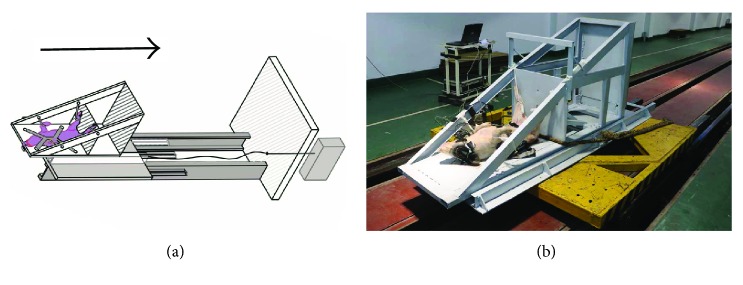
The schematic diagram and photograph of the installation. (a) Schematic diagram. The setup comprises a sled with three thin steel pipes, a horizontal seat assembly fixed on the carriage, rail, rigid wall, and traction control system. When the thin steel pipes collide with the rigid wall, the sled stops and the carriage continues to move forward and impact with the rigid wall, and finally the buttock of the pig experienced a high caudocephalad acceleration loaded from the seat. (b) The setup photograph. The pig lays in a supine position on the carriage, and the setup was ready for the CCI.

A modified deceleration sled was used to mimic the CCI to the animals.  Figure 1 illustrates the schematic diagram and photo of the experimental instruments. The setup consists of a horizontally orientated seat assembly, fixed on a carriage designed to slide with respect to the sled. When beginning the test, the sled and carriage were accelerated simultaneously to the configured velocity, and then the sled was stopped by the thin steel pipes as energy absorbers, fitted in front of the sled (with a thickness of 1.5 mm and a section of 80 mm × 80 mm). The carriage continued to move towards the rigid wall along the two rails of “C” shape and stopped by contacting with the rigid wall (Figure 1). A piece of hard rubber, mounted in the frontal of the wall, may avoid the destruction due to a high acceleration that resulted from the impact between the carriage and the rigid wall.

### 2.2. Animal Preparation

A total of 21 healthy Guizhou Congjiang adult minipigs, weighing 25–30 kg, either male or female, were obtained from the Experimental Animal Center of Daping Hospital, Third Military Medical University, Chongqing, China. The animals were housed in ventilated rooms and allowed to acclimatize to their surroundings for over 4 days before the test, and good physical conditions were kept for all the animals. All animals were fastened but free of water for 8 h prior to the tests. The animals were divided into randomly 3 groups: control group (*n* = 3), group I (*n* = 9), and group II (*n* = 9).

After premedication with an intramuscular injection of ketamine (30 mg/kg) and atropine (0.1 mg/kg), anesthesia was induced by xylazine hydrochloride (2 mg/kg, intramuscular injection) and maintained by pentobarbital (10 mg/kg/h, injecting through ear vein). The animals were tracheotomised with a tracheotomy cannula where respiratory rate was adjusted to obtain arterial PCO_2_ between 35 and 45 mmHg. Body temperature was maintained between 37°C and 39°C with a blanket. Cardiocirculatory and respiratory parameters were monitored continuously. Before the impact, all animals received an intravenous injection of 2 mg/kg carprofen to prevent pain. After stable anesthesia, artificial ventilation was shopped for the whole process.

**Figure 2:**
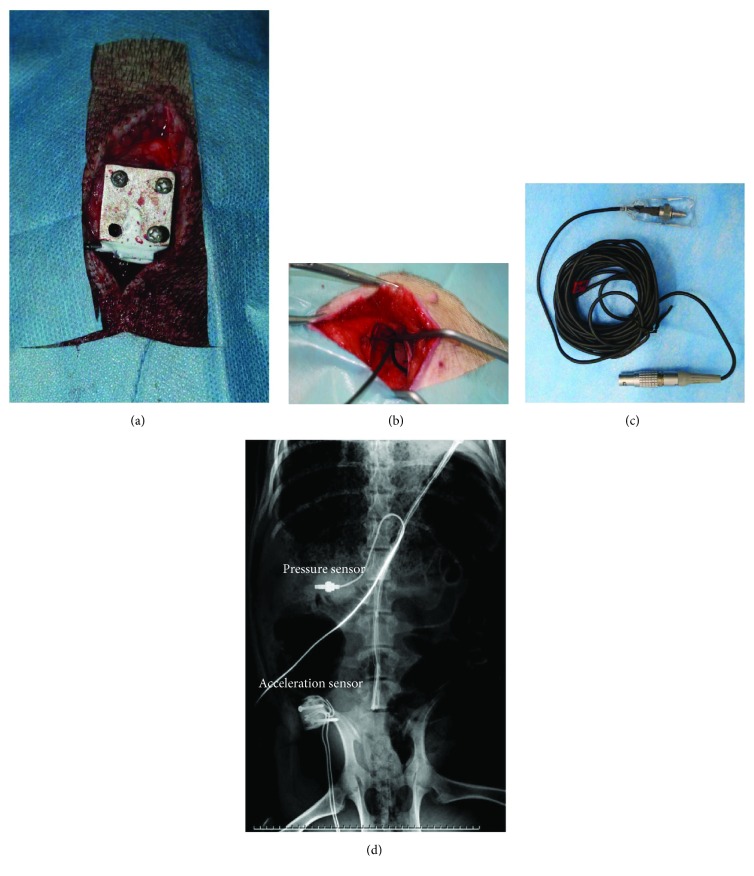
The installation of the sensors. (a) The operation photograph of the acceleration sensor installation. The sensor was glued to a metal block that was mounted on the pelvis via screws. (b) The operation photograph of the pressure sensor installation. The laparotomy was done, and the sensor was placed under the liver. (c) The photograph of the pressure sensor. The sensor was inserted into a balloon filled with liquid. (d) The X-ray photograph, in which the sensors were indicated with arrows.

Two sensors were used to record in vivo the mechanical response during the test. One was an acceleration sensor (7287-1-300, Endevco, USA; acceleration range: 0–2000 g), and the other was a pressure sensor (CYG41000, Dechen, China; pressure range 0–500 kpa). A metal block was mounted on the pelvis with several screws, and the acceleration sensor was glued to the block to measure the acceleration along the caudocephalad direction. The pressure sensor was inserted into a balloon filled with the liquid and placed under the liver to measure the abdomen pressure. The installation of the sensors is shown in Figure 2.

### 2.3. The Experimental Procedure

The anesthetized pig was fastened to seat with a 4-point hardness to avoid the throwing during experiencing the impact, in which the buttock of the subject contacted the rigid seat prior to the test, and the limbs were loosely fixed in the frame, mimicking a “sitting” posture (Figure 1), so that the loading may be acted on the buttock of the subject along the caudocephalad direction. Impact velocity of the sled was configured as 8 m/s in group I and 11 m/s in group II, respectively. The animals in the control group experienced the same test procedure in which the impact speed was defined as 0 m/s. When the experiment was initiated, the sled was accelerated from the starting position, running along the rail with a length of about 80.0 m, and stopped by the contact between the thin-wall pipes and rigid wall. The impact velocity of the sled was determined with a customer speed dosimeter. The whole course of the CCI was recorded by a high-speed video camera (Phantom V4.3, Vision Research), at 1000 frames per second.

### 2.4. Injury General Examination

Heart rate (HR), respiratory rate (RR), blood pressure (BP), and arterial blood gas analyses (ABGA, portable blood gas analyzer: i-STAT, Princeton NJ, USA) of all the subjects were recorded within 6 h postinjury at an interval of 1 h, in which the phase of 0 h referred to the immediate record after the test.

### 2.5. Injury Pathological Examination

Full-body CT scans were performed randomly for two pigs in group I and group II pre- and postinjury, to detect injury to the skeleton. The animals were sacrificed 6 h postinjury, and then considerable autopsies were carried out. The tissue at the injured location was obtained and prepared for microscopic observation, and the histopathological alternations were recorded. The autopsies were done immediately once the animals died. The injuries of the subjects were evaluated according to the Abbreviated Injury Scale 2005 (AIS-2005) [24], and the maximal AIS (MAIS) of each anatomical region was used to calculate the Injury Severity Score (ISS).

### 2.6. Statistical Analysis

ABGA and ISS were presented as mean ± SD and were analyzed using SPSS® 19.0 software. The differences of the acquired histopathological alternations in tested animals were tested using one-way analysis of variance (ANOVA). Comparison of AIS in groups I and II was conducted using chi-square test, and the ISS comparison between groups I and II was done using *t*-test. A value of *p* < 0.05 was considered statistically significant.

## 3. Results

**Figure 3:**
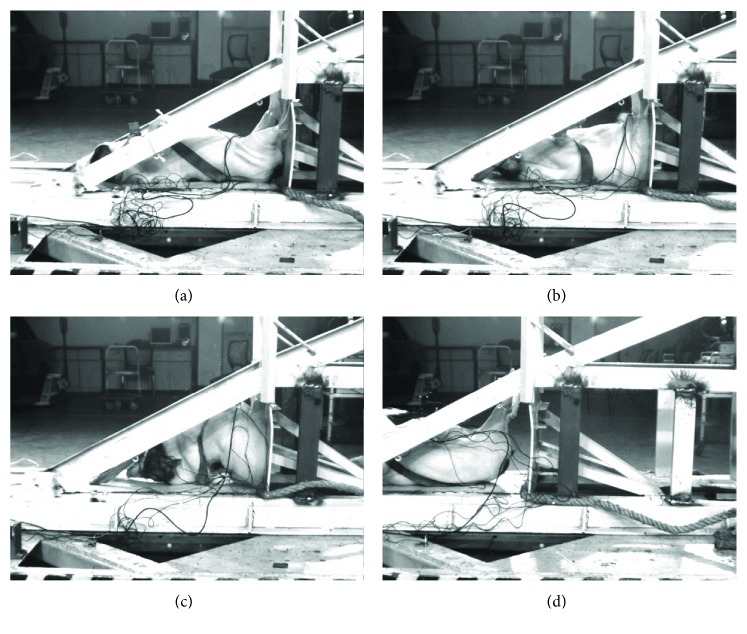
The kinematics of the pig during the CCI. (a) T0: the sled is hitting against the wall but the pig was motionless to the carriage. (b) T1: the pig showed maximal deformation of the abdomen due to severe compression of the torso along the caudocephalad direction. (c) T2: peak point of the spine bending. The maximal height (MH) indicated with the arrows refers to the distance between the peak point of the spine bending and the deck of carriage. (d) T3: the spine returned to normal form.

The determined impact speeds of groups I and II were 8.21 ± 0.13 m/s and 10.69 ± 0.41 m/s. The peak accelerations of the sled were 21.27 ± 1.70 g in group I and 34.07 ± 13.56 g in group II.  Figure 3 shows the kinematics of the subjects during crashing from a series of images derived from the high-speed camera, in which the maximal height (MH) was used to estimate the bending of the thoracolumbar spine, as shown in Figure 3(c). The MH in group II was significantly higher than that in group I (15.01 ± 0.98 cm versus 11.27 ± 1.67 cm, *p* < 0.05).

**Figure 4:**
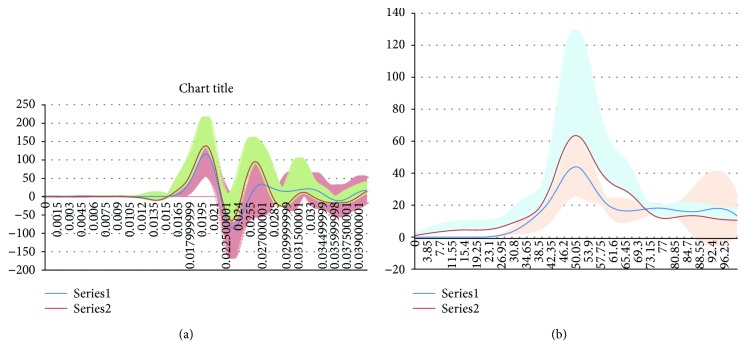
The corridor curves of the acceleration and pressure. The corridor of the acceleration (a) and the pressure (b) was showed, in which the acceleration and the pressure were indicated with different colors, with black referring to the data in group I and red in group II.

The peak acceleration recorded on the subject pelvis in group II was 139.13 ± 78.54 g, significantly higher than that in group I, 108.92 ± 58.87 g (*p* < 0.01), while the peak pressure of the abdomen in group II, 63.61 ± 65.83 kPa, was significantly higher than that in group I, 41.24 ± 16.89 kPa (*p* < 0.05).  Figure 4 shows the corridor curves of the caudocephalad accelerations and the abdomen internal pressure.

**Figure 5:**
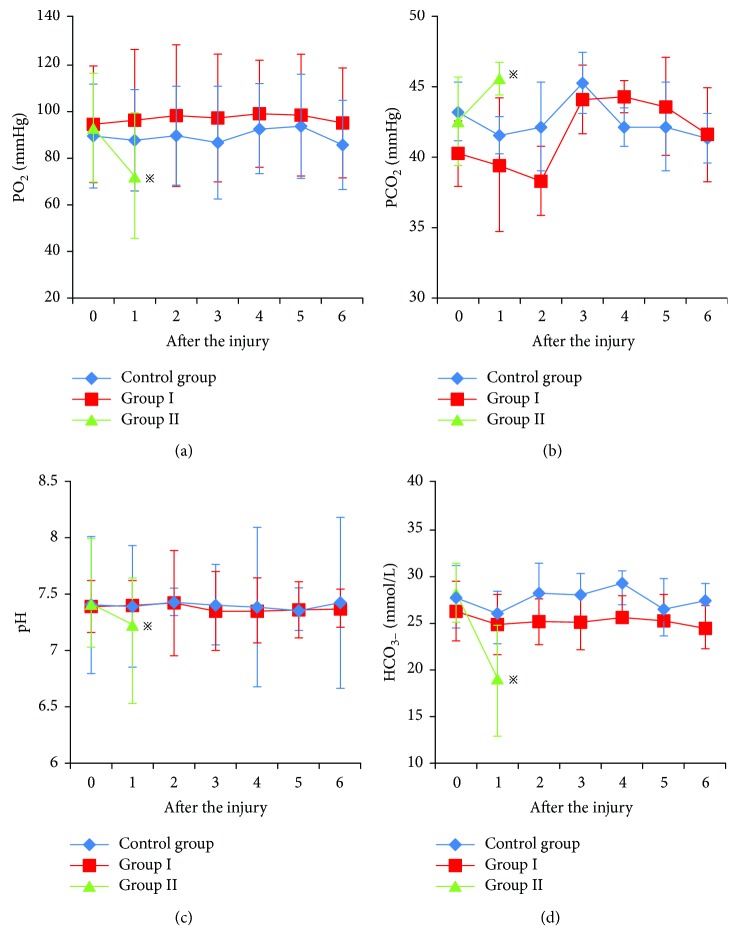
Arterial blood gas analysis. (a) PO_2_, (b) PCO_2_, (c) pH, and (d) HCO_3_ are detected in all three groups. ^※^Deterioration of ABGA (PCO_2_, PO_2_, pH, and HCO_3−_) at 1 h compared with baseline and control group, *p* < 0.05.

All the animals in group I survived, while in group II, 5 died of the mass bleeding within 2 h posttest, and 1 died immediately after the impact. Among all the subjects, HR and RR speeded up immediately after the impact and lasted for a few minutes. Subsequently, HR and RR in group I recovered to normal level, as compared to those in the control group, while HR and RR in group II kept at higher levels. BP in group I decreased rapidly after the impact and then remained stable at 40% of normal levels, in which BP in group II dropped to 10–20% of normal levels. Oxygenation parameters did not shift in the control group. The PCO_2_, PO_2_, pH, and HCO_3−_ in group I indicated a decreasing trend, as compared to those in the control group. In group II, the value of ABGA showed a deterioration at 1 h, as compared to that in the control group, but did not reach the criteria of respiratory failure (Figure 5).

**Figure 6:**
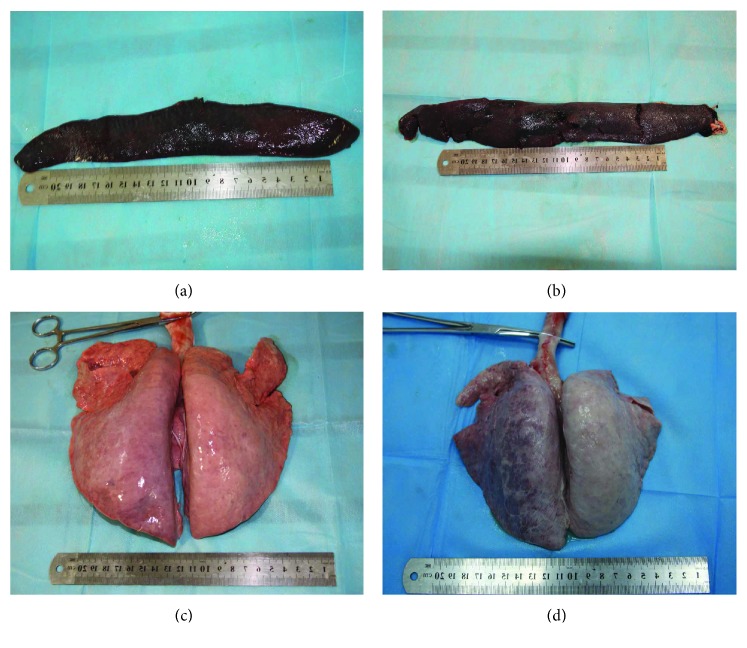
Typical injuries of the thoracoabdominal organs of the pigs that sustained CCI. (a) Ruptures of the spleen in group I, with 2-3 small wounds in the spleen indicated; (b) multiple wounds of the spleen in group II in which the wounds were more wider and deeper than those of group I; (c) there was no obvious damage to the lung of the pigs in group I after the impact; (d) diffuse hemorrhage of the lung of the pig in group II, in which the lamellar hemorrhage was observed.

**Figure 7:**
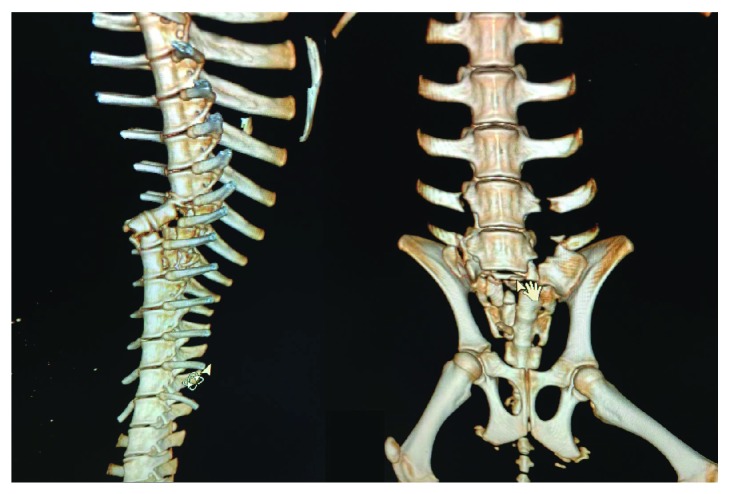
3D CT reconstruction of the spine and pelvic fractures.

**Figure 8:**
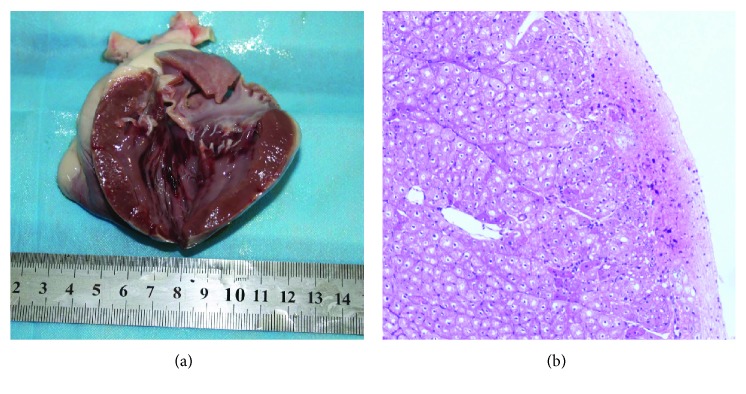
Subendocardial hemorrhage in gross and microscopic observation. (a) Subendocardial hemorrhage in gross observation in group I. (b) Subendocardial hemorrhage under microscopic observation in group I (HE ×100).

The common thoracoabdomen injuries from the autopsies included fractures, contusion, laceration, bleeding, and hemorrhage. The injured thoracoabdominal organs, that is, the spleen, lung, heart, and kidney, were observed commonly; however, the rate of subendocardial hemorrhage (SEH) in group I (4/9) was higher, as compared to that in group II (1/9). Furthermore, rib fractures and liver injuries have never been detected from the tested pigs.  Figure 6 shows the typical injuries to the thoracoabdomen organs, Figure 7 exhibits the fractures in the thoracolumbar spine and pelvis, and Figure 8 illustrates the heart injuries in gross and microscopic observation.

The MAIS of chest injuries was 4 in group I and 5 in group II, while 8 in group I and 9 in group II experienced the MAIS 5 abdomen injuries. There was a significant distribution discrepancy in MAIS between both groups (*p* < 0.05), as shown in Table 1. The ISS of group II, 52.71 ± 6.13, was significantly higher than that of group I, 26.67 ± 5.02 (*p* < 0.05).

## 4. Discussion

For the CCI-induced thoracoabdomen injuries, especially at a high loading rate, which frequently occurred in the military or civilian scenarios, the authors presumed that there may be two ways to transmit the vertical loading: one is the bone, and the other is the soft tissues, that is, thoracoabdomen muscles and organs, and the energy transmission may result in the injuries to the torso. Among the previously reported literature, most studies were done by carrying out experiments with a dummy or cadaver [16, 17, 20–22], in which the spine fractures have been validated from the published studies, while the response of the transmission along the soft tissue seldom was reported. In the present study, the CCI experiments were performed using adult minipigs at the different impact speeds, through which the thoracoabdomen injuries caused by the CCI and the response were studied.

The spine plays a key role in supporting the torso and transporting the loading along the vertical direction. The thoracolumbar spinal curvature changes from kyphotic to lordotic [25], while the thoracolumbar junction is particularly susceptible to fracture as it is under significant biomechanical stress due to the articulation of the relatively rigid thoracic segment, through its connections to the ribcage and sternum, with the more mobile lumbar region [26]. For example, it was reported that in aviation accidents, very few fractures occurred at the cranial and caudal levels, for example, 2% of fractures in the cervical spine, 78% of fractures in the thoracic spine, and 19% of fractures in the lumbosacral spine [27]. In the experiment, all subjects sustained the thoracolumbar fractures, and with an increase of the impact speed, the spine fractures became worse, affecting the stability of the spine. It can be concluded that the CCI-induced spine injuries (Figure 7) were associated with not only the compression but also the bending (Figure 3).

The review of Bailey et al. [28] suggested that among the experiments in studying pelvis and lower-extremity injuries using intact cadavers, the speed of a seat plate of a military vehicle subjected to the UBB may be up to about 12 m/s, while for the regulation of fall tests of a helicopter, the test speed was configured at 8 m/s and 12 m/s. In this study, we found the thoracoabdomen organs sustained severe injuries due to the vertical load transmission, while the severe and critical injuries could be reproduced by the CCI at the impact speed of 8 m/s and 11 m/s. Among the injuries, some remarkable characteristics in injury distribution and pattern for the injured thoracoabdomen organs were represented. In the experiment, the injuries of the thoracoabdomen organs, that is, the spleen, lung, heart, and kidney, were detected, and the injury patterns included contusion, laceration, and bleeding. The injuries observed in the study were coincident with those reported upon the autopsies for the victims involved in some accidents [29, 30]. However, some thoracoabdomen injuries, that is, rib fractures, aortic tears, cardiac rupture, and liver contusion, reported in the aviation accidents and falls [29, 30], were not reproduced in the current experiment. The authors suggest that among the autopsies for the victims, the injuries resulted from not only vertical but also horizontal. To the authors' viewpoint, therefore, the founding was valuable for the forensic worker to delineate the injury cause and distinguish the injuries induced by vertical, frontal, or lateral impact.

According to the study, it was concluded that for the injuries induced by the vertical loading on the buttock, the abdomen injuries were more severe as compared with the chest injuries. Our study showed that the mass bleeding due to the spleen laceration attributed to the death soon without any timely treatment, which means for the CCI injuries, the injury management for the control of bleeding is necessary to reduce the deaths. Kwon et al. [31] suggested that traumatic pelvic fracture patient prognosis needs to be improved through early diagnosis and prompt delivery of aggressive treatments based on rapid identification of abdominal solid organ injuries. However, previous pelvis trauma studies about bleeding control mostly focused on the injuries to the liver rather than the spleen, whereby numerous animal models were developed using pigs [32–39].

Among the abdomen injuries from horizontal impacts, liver injuries were detected frequently. Lau and Viano [40] considered that there were two regions of biomechanical response to blunt hepatic injury at the impact speeds of >12 m/s or ≤12 m/s. Some studies considered that the abdomen pressure may be an ideal predictor of liver injuries from horizontal loading [41–44]. From the current experiment, at the impact speed of 10.69 ± 0.41 m/s, the abdomen internal pressure was up to 63.61 ± 65.83 kPa, while the subjects in group I and group II did not experience liver injury. It may be concluded from the current study that the spleen is more vulnerable as compared to the liver for the CCI, and as a consequence, the prevention against spleen injuries also be paid a great attention to the CCI.

The lung injuries caused in road traffic accidents were considered traditionally to be associated with the impact speed and chest compression deflection, while in the present study, without any direct impact to the chest, lung injuries occurred frequently, so the authors presumed that the deceleration [45] and pressure changes on the chest [46] induced by the rapid diaphragmatic movement during high vertical loading may contribute to diffused lung injury (Figure 6). Our results showed the lung injuries may rapidly cause the deterioration of respiratory function in the critical injuries.

The CCI could result in heart injuries, that is, SEH, while a high rate of the SEH was found in group I (4/9, 44%), rather than in group II (1/9, 11%). The results suggest that the CCI contributed not only to primary SEH but the CCI-induced injuries may also lead to secondary SEH. Some previous studies concluded that the SEH may be related not only to the exposure to high acceleration [47] but also to brain trauma [48] and hemorrhagic shock [49]. Charaschaisri et al. [50] considered that even minor SEH might have an influence on cardiac function and might be involved in the mechanism of death. Therefore, besides injury prevention against heart injuries, for the injuries sustaining the CCI, the cardiac function should be also in monitor in injury management.

### 4.1. Limitations

Although the characteristics of the thoracoabdomen injuries induced by the CCI were addressed in the current study, some limitations still exist. Firstly, the CCI mimicking vertical impacts in the present study may ignore the influence of gravity. Secondly, the anatomical structure of pigs in the thoracoabdomen differs from human to some extent, especially for curvature of the spine. Thirdly, the pathological changes of minipigs were observed in the acute stage, while some researchers may concern the secondary injuries such as thermal injuries. Therefore, more detailed study regarding the vertical impact should be conducted.

## 5. Conclusion

Although the caudocephalad impacts (CCI) resulted in large numbers of injuries and deaths, little was known for the CCI-induced thoracoabdomen injuries. A total of 21 adult minipigs were used to carry out the CCI experiments to study the thoracoabdomen injuries, with a modified deceleration sled equipment at the configured speeds. In the present study, the kinematics and mechanical response of the tested animals were analyzed at the different impact speeds. The vital signs and arterial blood gas analysis (ABGA) of the animals were examined. The thoracoabdominal organs, such as the spleen, lung, heart, and spine, were injured frequently, but no liver injury was detected, and the common injuries included fractures, contusion, laceration, bleeding, and hemorrhage. The results presented here may be useful in forensic science, emergency management, and injury prevention.

## Figures and Tables

**Table 1:** The AIS distribution of the injuries of the animals.

Position	Group I AIS				Group II AIS			
	0	1 ~ 2	3 ~ 4	5 ~ 6	0	1 ~ 2	3 ~ 4	5 ~ 6
Head	—	—	0	0	—	—	0	0
Neck	9	0	0	0	9	0	0	0
Chest	7	1	1	0	1	0	8	0
Abdomen	0	0	8	1	0	0	0	9
Spine	0	9	0	0	0	0	3	6
Pelvis	0	0	9	0	0	0	0	9
