# Expression of Concern: Experimental Study of Thoracoabdominal Injuries Suffered from Caudocephalad Impacts Using Pigs

**DOI:** 10.1155/2019/4785406

**Published:** 2019-12-15

**Authors:** Applied Bionics and Biomechanics

*Applied Bionics and Biomechanics* would like to express concern with the article titled “Experimental Study of Thoracoabdominal Injuries Suffered from Caudocephalad Impacts Using Pigs” [1].

Concerns have been raised regarding the ethical standards of the reported research, which we feel require further investigation. These investigations will aim to determine whether the research adheres to the recommendations of the Guide for the Care and Use of Laboratory Animals of the National Institutes of Health, as stated in the manuscript.

The authors have been informed of this notice and the on-going investigation.
